# Climate Change Impacts on and Response Strategies for Kiwifruit Production: A Comprehensive Review

**DOI:** 10.3390/plants13172354

**Published:** 2024-08-23

**Authors:** Priyanka Rajan, Premkumar Natraj, Misun Kim, Mockhee Lee, Yeon Jin Jang, Young Jae Lee, Seong Cheol Kim

**Affiliations:** 1 Research Institute of Climate Change and Agriculture, National Institute of Horticultural and Herbal Science, Rural Development Administration, Jeju 63240, Republic of Korea; pri1995@korea.kr (P.R.); mkim2019@korea.kr (M.K.); mockey92@korea.kr (M.L.); ind03026@korea.kr (Y.J.J.); 2 College of Veterinary Medicine, Jeju National University, Jeju 63243, Republic of Korea; premkumar.n@jejunu.ac.kr (P.N.); yjlee3@jejunu.ac.kr (Y.J.L.)

**Keywords:** extreme weather events, mitigation strategies, kiwifruit cultivation, subtropical fruit

## Abstract

Climate change, a pressing global concern, poses significant challenges to agricultural systems worldwide. Among the myriad impacts of climate change, the cultivation of kiwifruit trees (*Actinidia* spp.) faces multifaceted challenges. In this review, we delve into the intricate effects of climate change on kiwifruit production, which span phenological shifts, distributional changes, physiological responses, and ecological interactions. Understanding these complexities is crucial for devising effective adaptation and mitigation strategies to safeguard kiwifruit production amidst climate variability. This review scrutinizes the influence of rising global temperatures, altered precipitation patterns, and a heightened frequency of extreme weather events on the regions where kiwifruits are cultivated. Additionally, it delves into the ramifications of changing climatic conditions on kiwifruit tree physiology, phenology, and susceptibility to pests and diseases. The economic and social repercussions of climate change on kiwifruit production, including yield losses, livelihood impacts, and market dynamics, are thoroughly examined. In response to these challenges, this review proposes tailored adaptation and mitigation strategies for kiwifruit cultivation. This includes breeding climate-resilient kiwifruit cultivars of the *Actinidia* species that could withstand drought and high temperatures. Additional measures would involve implementing sustainable farming practices like irrigation, mulching, rain shelters, and shade management, as well as conserving soil and water resources. Through an examination of the literature, this review showcases the existing innovative approaches for climate change adaptation in kiwifruit farming. It concludes with recommendations for future research directions aimed at promoting the sustainability and resilience of fruit production, particularly in the context of kiwifruit cultivation, amid a changing climate.

## 1. Introduction

Over the past decade, there has been a remarkable surge in the production and commercialization of tropical and subtropical fruits, particularly in regions with favorable climates, such as the subtropical and Mediterranean zones [1]. These regions offer ideal conditions for the cultivation of a diverse array of fruits, characterized by warm temperatures, ample sunshine, and sufficient moisture levels [2]. Subtropical fruits encompass a wide variety of species, each with its own unique characteristics and flavors. These fruits are typically rich in essential nutrients, vitamins, and antioxidants, making them not only delicious but also highly nutritious choices for consumers [3,4,5]. These fruits are often characterized by their vibrant colors, juicy flesh, and exotic flavors, making them popular ingredients in a wide range of culinary dishes, beverages, and desserts [6,7,8]. Subtropical fruits thrive in slightly cooler climates than tropical fruits, but still require relatively warm temperatures to grow [9]. Some common subtropical fruits include citrus fruits like oranges, lemons, and grapefruits, as well as kiwifruits, figs, and persimmons [10]. The increasing popularity of subtropical fruits can be attributed to several factors, including growing consumer interest in exotic and healthy food options, advancements in agricultural technology and practices, and expanded global trade networks that facilitate the transportation and distribution of these fruits to markets around the world [5,11,12,13,14,15]. Overall, subtropical fruits play a significant role in global agriculture and food systems, providing consumers with a diverse range of nutritious and flavorful options to enjoy year-round. Their cultivation and commercialization contribute to the economic development and food security of many regions, while also promoting sustainable farming practices and environmental stewardship.

Kiwifruit, also known as the Chinese gooseberry, is a small, nutrient-dense fruit native to China [16]. It is renowned for its vibrant gold and red flesh speckled with tiny black seeds and its tangy–sweet flavor [17]. Kiwifruit is rich in essential nutrients, such as vitamin C, vitamin K, vitamin E, potassium, and fiber, making it a popular choice for health-conscious consumers [18]. The commercial kiwifruit, belonging to the genus *Actinidia*, encompasses varieties such as *Actinidia chinensis* var. *deliciosa* (green-fleshed kiwifruit), *A. chinensis* var. *chinensis* (yellow fleshed), and the kiwi berry *A. arguta* [19]. Bardi noted the preference of *A. chinensis* var. *deliciosa* for cool climates and higher altitudes [20]. The global kiwifruit industry has largely depended on the green-fleshed *A. deliciosa* cultivar, known as ‘Hayward’, for its advancement and expansion [21]. Over the years, the global production of kiwifruit has expanded, resulting in more land being dedicated to kiwifruit cultivation. In 1970, less than 1000 hectares were used for kiwifruit farming outside of China, but by 2010, this had increased to over 160,000 hectares globally [22]. The ‘Hayward’ fuzzy kiwifruit (*A. deliciosa*) is the predominant cultivar, constituting 50% of global cultivation and 90–95% of international trade [23]. While production reaches 1.8 million tons annually, kiwifruit represents just 0.2–0.3% of total fresh fruit output [24]. Despite its minor status, kiwifruit ranks sixth in marketable gross production, with China, Italy, New Zealand, Chile, and Greece as the key producers [23,25].

Climate change denotes persistent shifts in weather patterns, extending from the tropical regions to polar areas. It constitutes a ubiquitous global threat that imposes stress across diverse sectors [26]. Among the sectors most susceptible to the effects of climate change, agriculture stands out, having grappled with myriad challenges in recent times [27]. Given that the climate exerts a pivotal influence on delineating the geographical distribution of various plant species, agricultural production worldwide has been significantly affected by climate change [28,29]. The predominant drivers of these challenges are alterations in the mean temperatures, shifts in the minimum and maximum temperature thresholds, changes in rainfall patterns and amounts, and the emergence of biotic threats, such as pests and microorganisms. These factors, collectively, jeopardize plants’ abilities to adapt and survive in the face of evolving environmental conditions [30,31,32,33,34]. In recent years, regions renowned for their subtropical and Mediterranean climates, ideal for kiwifruit cultivation, have experienced notable shifts in weather patterns attributable to climate changes [35]. These changes include increasing temperatures, especially during the summer months, and prolonged periods of drought interspersed with heavy rainfall [36]. These alterations have significantly impacted the feasibility and efficiency of agricultural operations in these areas.

This review endeavors to furnish a thorough examination of the present state of kiwifruit cultivation in subtropical locales, placing particular emphasis on the impediments arising from climate change. Through a meticulous analysis of recent research discoveries and technological innovations, this paper aims to expound upon the methodologies for alleviating the detrimental impacts of climate change on kiwifruit farming. Moreover, it delves into the pioneering methodologies and optimal practices that could bolster the resilience and sustainability of kiwifruit production mechanisms amidst the dynamic backdrop of changing climatic circumstances.

## 2. Climate Change and Its Manifestations in Subtropical Regions

### 2.1. Global Temperature Rise and Its Impact on Kiwifruit Production

To systematically illustrate the diverse impacts of climate change on kiwifruit production, Table 1 summarizes the key phenomena, their specific effects, affected locations, and insights from various studies. This consolidated view offers a comprehensive understanding of how climate variables are shaping current and future kiwifruit cultivation practices. Adequate winter chilling is crucial for initiating dormancy release and promoting the abundant spring flowering of kiwifruit. However, with the steady rise in global and New Zealand average temperatures, there is an increasing risk of insufficient winter chilling. Climate change, particularly temperature fluctuations, impacts the feasibility of cultivating *A. chinensis* and *A. deliciosa* cultivars in Te Puke, New Zealand, leading to declining production viability and rendering Te Puke non-viable for kiwifruit cultivation by the century’s end [37]. In recent years, Kiwifruit Early Decline Syndrome (KEDS) has become increasingly severe and widespread, adversely impacting kiwifruit orchards globally and significantly affecting the economic sustainability of rural farms. This syndrome, characterized by the sudden collapse and death of affected plants, is believed to be a consequence of climate change-induced phenomena. High-temperature stress directly affects kiwifruit plant growth, physiology, and the allocation of photoassimilates, while also indirectly leading to hypoxic soil conditions [20,36]. A previous study highlighted the impact of climate change on kiwifruit farming in Kastamonu, Turkey. The findings from a survey of 65 producers revealed that the seasonal air temperature in their region had increased, emphasizing the importance of adaptation measures [38]. The research indicated that high temperatures severely impair kiwifruit cultivars’ photosynthetic rate, leading to irreversible leaf damage at 52 °C, underscoring the critical impact of heat stress on kiwifruit productivity and necessitating the appropriate cultivar selection for climate resilience [39]. Elevated temperatures (3–5 °C above ambient) during the key stages of kiwifruit development shift resources towards vegetative growth, significantly reducing fruit carbohydrate and vitamin C levels. Additionally, heat exposure negatively impacts vine growth, flowering, and fruit ripening in subsequent seasons. This underscores the critical importance of temperature management for preserving fruit quality and overall vine health [40].

### 2.2. Frosting and Its Impact on Kiwifruit-Producing Regions

Climate change is amplifying the risks to kiwifruit cultivation, particularly evident in the Boseong, Jeollanam-do provinces of Korea, where the threat of spring frost damage is on the rise. This increased risk is attributed to the projected overlapping of vulnerable kiwifruit phenophases around 2100. Frost significantly impacts kiwifruit production, as seen with the earlier last-frost dates and accelerated bud burst due to climate change. Consequently, the risk of spring frost damage is escalating. In light of these findings, urgent adaptive measures are required to safeguard kiwifruit production against the worsening threat of frost damage driven by climate change. By informing policymakers and stakeholders, these strategies could better prepare communities to mitigate the effects of climate change and sustain kiwifruit production amidst evolving environmental challenges [41]. A chilling temperature of below 7 °C for a period of 700–1000 h plays a crucial role in the flowering and fruiting of kiwifruit. A study involving respondents revealed the various effects of high-intensity cold on kiwifruit plants. Specifically, the shedding of old leaves, late maturity and harvest, and small-sized fruits were reported. Additionally, fruit hardening was noted. It was recommended to avoid freezing temperatures and to protect young kiwifruit plants from exposure to very cold and freezing temperatures. Overall, these findings underscore the importance of proper chilling temperatures for optimal kiwifruit flowering and fruiting [42].

### 2.3. Changes in Extreme Weather Events: Droughts, Floods, and Typhoons

Changes in extreme weather events, including droughts, floods, and typhoons, have had a profound impact on kiwifruit production. Kiwifruit, native to the mountains of southern China, has evolved to grow in high-humidity environments with regular annual rainfall [24]. In New Zealand, it is a significant commercial crop, primarily cultivated in the Bay of Plenty, but also in a region extending from Kerikeri in the north to Nelson in the south [43]. With the extensive planting of gold kiwifruit (*A. chinensis* var. *chinensis* ‘HORT16A’) in areas with a low soil water holding capacity and variable rainfall, understanding its water stress physiology is crucial. A study observed that drought conditions can significantly impact kiwifruit photosynthesis by inducing elevated leaf temperatures, leading to limitations on its photosynthetic processes. Studies have demonstrated constraints on photosynthesis in *A. deliciosa* at leaf temperatures above 25 °C. The reduction in the photosynthetic rate during drought phases may be attributed to disruptions of the photosynthetic mechanisms under elevated leaf temperatures. Effective field management practices, such as irrigation, are essential to mitigate these adverse effects on kiwifruit production. Utilizing irrigation water to cool the canopy can help prevent leaf heating and potential damage, ensuring the sustainability of kiwifruit cultivation in drought-prone environments [43].

A study demonstrated that droughts and floods have significant adverse effects on kiwifruit production, reducing both yield and fruit quality. The study observed that abscisic acid (ABA) plays a crucial role in kiwifruit’s response to water stress, with increased levels during drought and decreased levels during flooding. Understanding the molecular mechanisms underlying kiwifruit’s response to water stress is essential for developing strategies with which to increase resilience and productivity in the face of climate change [44].

Climate change-induced extreme weather events, such as intense storms and typhoons, exacerbate plant hypoxia, primarily through soil waterlogging and flooding. This poses a significant threat to global agricultural productivity [45]. Soil water saturation is escalating due to more frequent extreme weather events and shifts in seasonal patterns [57]. In numerous agricultural regions worldwide, escalating rainfall patterns linked to climate change have led to soil water accumulation, resulting in waterlogging [58,59]. Approximately 16% of cultivated land globally experiences waterlogging, causing a 20% decrease in yield [60]. Regions like Southeast China, where kiwifruit cultivation is prominent, suffer from excess rainfall during the summer rainy season [61].

Moreover, a study observed that strong wind, particularly cyclonic wind, adversely affects various stages of kiwifruit growth. It damages the entire plant and its branches, displaces vines, and causes significant orchard damage. During the fruit-setting stage, it disrupts fruit setting and bud dropping. During the flowering stage, it results in low pollination rates, branch breakage, and flower drop. It also causes fruit drop, spots on fruit, and may require premature harvesting. Gentle wind flow is crucial for the optimal vegetative and reproductive phases of kiwifruit plants [42]. These extreme weather events pose significant challenges to kiwifruit growers, necessitating adaptive strategies, such as improved irrigation techniques, soil management practices, and resilient orchard designs, to mitigate their adverse effects on kiwifruit production.

## 3. Effects of Climate Change on Kiwifruit Trees

### 3.1. Phenological Changes: Flowering, Fruiting, and Ripening

As we delve into the multifaceted impacts of climate change on kiwifruit production, it becomes clear that these effects are both varied and significant. To provide a structured overview, Table 1 categorizes the effects of climate change, detailing their specific impacts on kiwifruit production across various locations and summarizing the pertinent findings from recent studies. This table serves as a vital resource for understanding the nuanced interactions between climatic factors and kiwifruit cultivation, underscoring the urgent need for adaptive strategies in agricultural practices. In New Zealand, the period from May to July is highlighted as critical for ‘Hayward’ kiwifruit production, with winter temperatures influencing bud dormancy release and subsequent flowering [62]. The concept of “winter chilling” is crucial, impacting the flower quantity, timing, and compactness of the flowering period [63]. Te Puke township in New Zealand’s Bay of Plenty region serves as the hub of the country’s kiwifruit industry, where over 90% of production is located, primarily focusing on the ‘Hayward’ cultivar, renowned globally for its commercial success [35]. Historically, the region’s climate and soil conditions have been conducive for kiwifruit cultivation, characterized by ideal temperature regimes, with warmer summer days of 23–25 °C, good sunshine hours (>2000 h), and favorable rainfall throughout the year (1300 mm) [64,65]. A recent modeling approach underscores a concerning trend: ‘Hayward’ kiwifruit production viability in Te Puke is projected to decline steadily, becoming consistently marginal by the 2030s, and predominantly poor by the century’s end. This reduction in viability is primarily attributed to insufficient winter chilling, as temperatures rise due to climate change [37]. Overall, this analysis highlights the vulnerability of ‘Hayward’ kiwifruit production in Te Puke to climate change and suggests potential shifts in cultivation patterns to more favorable regions within New Zealand. Another study highlights the significant impact of climate change on the phenological patterns of kiwifruit cultivation in Korea, particularly focusing on the flowering stage of the ‘Haegeum’ cultivar in the Jeallanam-do province. Utilizing a ‘Chill-day’ model and six global climate models, the research underscores the uncertainty of climate change scenarios. It predicts a notable increase in the flowering days for ‘Haegeum’, potentially exceeding 10 days compared to present conditions, with variations among the individual climate models. Moreover, the study suggests a northward expansion of the flowering period, reaching into neighboring provinces. These findings underscore the importance of considering climate change implications for agricultural practices, and highlight the need for further research in phenology modeling to comprehensively assess local impacts [46].

Warm winters, occurring during kiwifruit’s dormant period, have a pronounced effect on the fruiting of kiwifruit plants. These variations directly influence the release of bud dormancy, affecting the timing and abundance of flowering. Consequently, temperature plays a crucial role in determining the yield of kiwifruits, by influencing the number of flowers produced per bud and the overall timing of flowering [37,66]. A study observed that temperature variations of 2–5 °C above the ambient temperature during the growing season can have significant effects on the quality attributes of kiwifruit. These attributes, including fruit weight, dry matter concentration, and soluble solids concentration, are crucial measures of the fruit quality of kiwifruit. Specifically, spring warmth accelerated flowering by 17 days, shoot growth by 6 mm d^−1^ °C^−1^, and increased fruit size by 3.5 g, while summer heat hindered fruit growth, dry matter accumulation, and firmness [67].

### 3.2. Shift in Distribution Ranges and Suitable Growing Regions

Global warming has enabled the cultivation of tropical crops in Korea that were previously unsuitable for its climate, leading to an expansion of their cultivation areas post-introduction [47]. As temperatures rise, certain crops will find new suitable regions for cultivation, leading to shifts in cultivated crop species [68]. In regions where high winds were once uncommon, such as the southern parts of Korea, the cultivation of kiwifruits has become increasingly popular. For instance, the main production regions of kiwifruit, a relatively new crop to Korea, were primarily in Jeju Island and select south-coastal regions, like Mokpo, Haenam, and Goheung in the Jeallanam-do province, of the Korean peninsula in 1990. By 2005, this cultivation area had expanded to include Sacheon (Gyeongsangnam-do province) and the entire region of Jeju Island [47]. In Korea, the area dedicated to kiwifruit cultivation expanded from 1122 ha in 2010 to 1335.2 ha in 2022. This rapid expansion over a mere 15-year period can be attributed not only to increased consumption, but also to the expansion of the warm regions suitable for cultivating kiwifruits due to global warming. Another research study indicated that due to global warming, the suitable geographic areas for kiwifruit cultivation in Jeju Island would transition from the coastal regions to elevated mountain areas, with an estimated shift of about 250 m in altitude [69].

### 3.3. Impact on Plant Physiology: Photosynthesis, Respiration, and Water Stress

Climate change has a profound impact on kiwifruit trees, influencing their physiology and overall well-being. The photosynthesis of kiwifruit plants is particularly susceptible to the challenges posed by climate change, as they are highly sensitive to environmental variables like temperature, humidity, and water availability [48,49]. As temperatures rise and humidity levels increase, kiwifruit plants may experience stress, leading to reduced photosynthetic efficiency [39]. Moreover, alterations to precipitation patterns can disrupt water availability, with drought conditions limiting water for photosynthesis, and waterlogging from heavy rainfall impeding root function [59,70]. The kiwifruit plant’s susceptibility to high-temperature and humidity stresses further complicates matters, affecting both its photosynthetic processes and fruit development. A recent study demonstrated that the close relationship between the transpiration rate and stomatal conductance underscores the plant’s responsiveness to environmental changes [39]. To mitigate these impacts, careful water management and adaptation strategies are essential to maintain optimal photosynthetic activity, and to ensure the health and productivity of kiwifruit cultivation amidst changing climatic conditions. In addition, recent research has highlighted the impact of rising temperatures, attributed to climate change, on the respiration rates of kiwifruit trees. Specifically, a recent study elucidated the significant effects of extreme temperatures on the respiration process in yellow-fleshed tetraploid kiwifruit (*A. chinensis*). The study identified 44.5 °C as the threshold temperature for respiration in *A. chinensis*. Beyond this point, irreversible leaf damage occurs, especially when temperatures exceed 52 °C. This damage likely stems from the excessive metabolic activity and stress caused by the extreme heat, leading to physiological disruptions and structural impairments of the plant tissues [39]. Furthermore, changes in precipitation patterns, including altered timing, intensity, and distribution of rainfall, can result in water stress in kiwifruit trees. Drought conditions can limit water availability for photosynthesis, ultimately affecting kiwifruit production [70]. Conversely, an increased frequency and intensity of rainfall events may cause soil saturation and waterlogging, negatively impacting root health and function [71].

### 3.4. Increased Susceptibility to Pests and Diseases

Climate change has the potential to induce shifts in the distribution and prevalence of existing insect pests and diseases, as well as heighten the likelihood of new pests emerging. It has exerted significant influence on the distribution patterns of various species, with future climate projections expected to further alter their habitats, ranges, and distributions [72,73,74]. Climate change significantly impacts the distribution and severity of plant diseases, posing a critical threat to agricultural productivity [75]. Changes in temperature, moisture, humidity, and seasonal variations directly influence the spread and abundance of plant pathogens [76]. Rising temperatures, altered precipitation patterns, and an increased frequency of extreme weather events create favorable conditions for disease development, and exacerbate the existing challenges to disease management [77]. These changes have precipitated shifts in the geographic extent and temporal dynamics of plant diseases, leading to alterations in their distribution, occurrence patterns, epidemiology, and population structures [78].

*Alternaria alternata*, *Botrytis cinerea*, *Cladosporium cladosporioides*, *Diaporthe* spp., *Nigrospora oryzae*, and *Trichothecium roseum* are all major pathogens of kiwifruit that decrease the quality of kiwifruit. Bacterial canker, attributed to *Pseudomonas syringae* pv. *actinidiae* (Psa), is the most destructive disease for kiwifruit worldwide, and imposes significant harm on global kiwifruit production, leading to considerable economic losses and plant fatalities [50,51,52,53,54]. Its symptoms encompass watery lesions on the twigs, trunks, leaves, and flowers, progressing to necrosis and wilting [79]. Initially identified in Japan in 1984, Psa has since spread to major kiwifruit-producing countries, such as China, causing widespread devastation [80]. Acknowledged as a substantial threat to the industry, Psa is classified as a quarantine organism, prompting unified endeavors to curtail its dissemination and alleviate its repercussions [81]. The escalating temperatures associated with climate change create favorable conditions for the proliferation and virulence of pathogens like Psa, exacerbating bacterial canker in kiwifruit [55]. A recent study underscored that the critical climate variables influencing Psa suitability include the annual mean temperature and annual precipitation, with excessive rainfall (>1200 mm/year) hindering the establishment of Psa [56].

Early kiwifruit decline, first observed in New Zealand after cyclone-induced flooding, stems from the plant’s susceptibility to root waterlogging and soil anoxia. While pathogenic microorganisms were found in the affected plants, they were deemed secondary to the waterlogging-induced weakening [36]. This highlights the fact that cyclones, particularly those causing heavy and prolonged flooding, can indirectly contribute to the survival and proliferation of pests and pathogens in kiwifruit orchards. Excess water can create conditions favorable for the growth and spread of various pests and pathogens [45,82].

## 4. Economic and Social Implications for Kiwifruit Production

### 4.1. Losses in Kiwifruit Production and Yield

Losses in kiwifruit production and yield can have far-reaching consequences, affecting both economic stability and food security. Kiwifruit cultivation is a significant economic activity in many regions around the world, contributing to the livelihoods of farmers and supporting agricultural industries. The New Zealand kiwifruit industry has experienced remarkable export success over the past three decades, despite facing typical market fluctuations and challenges. Revealed comparative advantage analyses have indicated that New Zealand possesses a notably high comparative advantage in kiwifruit production [83]. The industry has a substantial potential to contribute to the regional growth goals of the government, particularly in the Bay of Plenty (BOP), a significant horticultural region in New Zealand. With a population of 321,000, comprising 7% of the total national population, the BOP region contributes 5.7% to New Zealand’s GDP, and employs 6.6% of its workforce [84]. In 2010, both China (480,000 MT) and Italy (450,049 MT) had already surpassed New Zealand (372,833 MT) in kiwifruit production, yet the kiwifruit industry’s contribution to New Zealand’s GDP remained higher compared to that of Italy or China [22,52,85]. Various factors, including adverse weather, pests, diseases, and market fluctuations, can reduce kiwifruit production and yield, as exemplified by the Psa outbreak that led to substantial export losses. The Psa outbreak in New Zealand had a significant economic impact, with up to NZD 930 million in lost exports by 2014. This led to income loss and declining orchard values for growers, resulting in an overall equity loss of potentially NZD 2 billion [52]. Losses in kiwifruit production reduced availability, raising prices and placing a strain on both producers and consumers [86].

Climate change poses significant challenges to the kiwifruit industry, particularly in regions like the Kaituna catchment in New Zealand’s Bay of Plenty [87]. An increased frequency and intensity of extreme weather events, such as droughts and floods, can disrupt kiwifruit production by affecting both fruit quality and quantity [88]. Additionally, rising temperatures and altered precipitation patterns may shift optimal growing conditions for kiwifruit, potentially impacting yields and necessitating changes in cultivation practices [89]. In conclusion, climate change presents economic and social implications for kiwifruit production, with increased extreme weather events and shifting growing conditions threatening both fruit quality and quantity, necessitating adaptation measures, and potentially impacting livelihoods and agricultural practices. Addressing these losses is crucial for farmers’ economic well-being and the stability of national economies, given kiwifruit’s contribution to GDP and agricultural sectors globally.

### 4.2. Impacts on Livelihoods of Kiwifruit Farmers and Agricultural Communities

Kiwifruit farmers and their communities are already grappling with significant challenges due to diminished yields, such as financial stress, mounting debt, and poverty [90]. Entire regions that are reliant on kiwifruit cultivation are witnessing social disruptions, including eroded social cohesion and increased migration, as individuals seek alternative livelihoods [91,92]. These adversities precipitate labor shortages, reduced access to essential services, and the erosion of cultural heritage linked to kiwifruit farming traditions [93]. Furthermore, climate change exacerbates these detrimental impacts by further curtailing kiwifruit production. Previous findings have indicated that farmers’ fight against climate change has negatively impacted kiwi yield at the 95% confidence level [38]. Therefore, the negative effects of climate change further exacerbate these issues, making it imperative to address climate resilience in kiwifruit farming to safeguard these communities’ futures.

### 4.3. Market Fluctuations and Global Trade Dynamics of Kiwifruit

The market fluctuations and global trade dynamics of kiwifruit further compound the challenges faced by producers. Kiwifruit is a major player in global trade, with significant exports originating from countries like New Zealand, Italy, and Chile [22,94]. Together, these nations account for roughly 80% of kiwifruit production outside of China [94]. Changes in demand, trade policies, and international competition can impact the profitability of kiwifruit farming enterprises. For example, shifts in consumer preferences towards other fruits or changes in trade agreements can affect the demand for kiwifruit and its market price [83].

Above all, climate change exacerbates the impact of market fluctuations on kiwifruit growers by compounding financial risks, such as falling prices, declining demand, and high exchange rates. This “double exposure” scenario means that even with rising fruit prices, high exchange rates can reduce growers’ returns. Compliance with overseas standards like GLOBALGAP adds costs and management burdens, increasing financial strain. Additionally, global market competition from lower-cost producers creates price volatility. New Zealand kiwifruit commands a premium for its high quality, but this is vulnerable to market changes. Maintaining this premium is crucial, as losing it could severely impact growers already facing climate and market challenges [35,95,96]. These market dynamics introduce uncertainty for kiwifruit farmers, challenging their ability to plan and invest in their operations. Furthermore, small-scale farmers in developing nations may encounter difficulties accessing global markets and competing with larger producers, exacerbating inequalities in the kiwifruit trade.

## 5. Adaptation and Mitigation Strategies for Kiwifruit Cultivation

### 5.1. Breeding and Selection of Climate-Resilient Kiwifruit Cultivars

**Figure 1:**
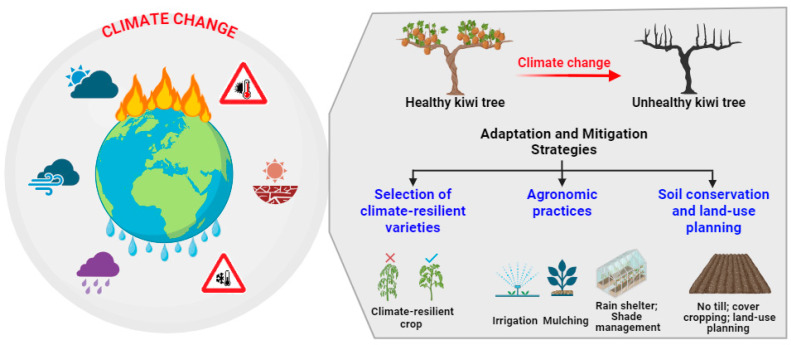
Adaptation and mitigation strategies for kiwifruit cultivation in response to climate change.

Meteorological disasters significantly impact various phases of kiwifruit growth. Frost damage during tree dormancy and germination phases, summer drought during fruit development, high-temperature sunburn during fruit maturity, and continuous rain disasters during fruit maturity are particularly influential [96]. Table 2 and Figure 1 highlight the various adaptation and mitigation strategies employed to address the impacts of climate change on kiwifruit cultivation. In response to the challenges posed by meteorological disasters to kiwifruit cultivation, one key adaptation strategy involves the breeding and selection of climate-resilient kiwifruit cultivars. Climate resilience refers to the ability of a socio-ecological system to endure and sustain its functionality amidst the external pressures induced by climate change. This encompasses two key aspects: firstly, a system’s capacity to absorb such stresses while retaining its essential functions, and secondly, its capability to adapt, restructure, and progress towards more advantageous states that enhance sustainability and readiness for forthcoming climate challenges [97,98,99]. By focusing on traits such as heat tolerance, drought resistance, and disease resilience, breeders can develop kiwifruit cultivars better suited to withstand extreme weather events. A study examined the impact of greenhouse conditions on photosynthetic factors in various kiwifruit species and cultivars, revealing their resilience and adaptive responses to abiotic stressors, such as high temperatures. These insights into their physiological adjustments and species-specific differences contributed to an understanding of kiwifruit plant resilience in changing climates [39]. A study investigated the drought stress tolerance mechanisms of different species of kiwifruit. The study suggested that both *A. arguta* and *A. eriantha* exhibit drought stress tolerance, highlighting their potential resilience to water scarcity conditions. This finding underscores the suitability of these species for cultivation in regions prone to drought, offering opportunities for sustainable kiwifruit production in challenging environments [100]. Through rigorous selection processes and advanced breeding techniques, including marker-assisted selection and genomic analysis, breeders could expedite the development of cultivars that exhibit enhanced resilience to meteorological stressors [101,102,103]. These climate-resilient kiwifruit cultivars could not only reduce the susceptibility of crops to weather-related damage, but also contribute to the overall sustainability and stability of kiwifruit production systems. A previous study elucidated the molecular mechanisms underlying high-temperature tolerance in kiwifruit, identifying 36 and 41 heat shock transcription factor (Hsf) genes in the *A. chinensis* and *A. eriantha* genomes, respectively. The analysis revealed the Hsf gene expression patterns linked to fruit ripening and stress responses, with the subcellular localization indicating a nuclear and cytoplasmic presence. Notably, AcHsfA2a emerged as a key player in the high-temperature stress response, offering insights for breeding heat-stress-resistant kiwifruit cultivars [104]. In conclusion, the integration of climate-resilient breeding strategies and molecular insights into high-temperature tolerance presents a promising pathway for enhancing kiwifruit production sustainability and resilience against meteorological disasters.

### 5.2. Agronomic Practices: Irrigation, Mulching, Rain-Shelter Cultivation System, and Shade Management for Kiwifruit Orchards

Climate change is altering the growing conditions in kiwifruit orchards, leading to challenges such as water scarcity, flooding, heat stress, and increased sun exposure [126]. To address these issues, optimizing the management of kiwifruit orchards through agronomic practices is essential for ensuring the resilience and productivity of kiwifruit cultivation. This involves implementing strategies, such as irrigation management, mulching, rain-shelter cultivation systems, and shade management, to address the key challenges and enhance the sustainability of kiwifruit production [127,128,129]. By focusing on these practices, growers can effectively manage water resources, promote soil health, and mitigate the impacts of environmental stressors on kiwifruit plants, ultimately contributing to the resilience and success of kiwifruit orchards [130].

Firstly, irrigation management is crucial for providing kiwifruit plants with adequate water throughout their growth stages, especially during the fruit expansion stage, which is an important period for the fruit’s water requirements. Drip and sprinkler irrigation, a commonly used method, delivers water directly to the root zone, minimizing water loss through evaporation and runoff [105]. It is essential to schedule irrigation based on factors, such as soil moisture levels, weather conditions, and the plant growth stage, to prevent both water stress and waterlogging [106].

Mulching is another key practice that offers numerous benefits to kiwifruit orchards. By applying organic materials, like straw or wood chips or synthetic mulch films, around the base of kiwifruit plants, soil moisture is conserved, weed growth is suppressed, soil temperatures are moderated, and erosion is prevented [107]. A previous study showed that a dual mulching treatment resulted in the highest kiwifruit yield per plant, with an increase of 14.9% compared to that of bare land without mulching [107]. In response to challenges like seasonal drought and high temperatures that impact kiwifruit yield and quality in South China, a recent study explored mulching methods to enhance orchard soil conditions. The field experiments compared six mulching treatments, showing significant improvements in soil moisture retention and temperature regulation. Mulching, especially weed-proof mulching combined with white mulching, increased soil moisture by up to 2.9% and decreased soil temperature by 0.8–2.5 °C, resulting in enhanced fruit yield and quality. Notably, the weed-proof mulching treatment demonstrated the most pronounced effects, making it the preferred method for orchard management in the region [108].

Providing adequate shelter is essential to protect kiwifruit flowers from heavy rainfall. Rain-shelter cultivation systems have been widely adopted in kiwifruit cultivation to mitigate the risks of diminished yield and fruit quality [109]. A recent study demonstrated that deficit drip irrigation under rain-shelter cultivation improved kiwifruit quality in the humid area of South China. The study showed that deficit drip irrigation improved the fruit quality and water productivity of kiwifruit while maintaining the yield, which increased the total soluble solids, titratable acidity, vitamin C, and water productivity by 9.1, 6.1, 19.2, and 4.6%, respectively [109]. Additionally, another study found that the incidence of disease in kiwifruit was significantly lower under a rain-sheltering system compared to an open-shelter cultivation system. This reduction in disease was attributed to an increase in bacterial and fungal taxa that act as biocontrol agents [110,111]. In conclusion, the adoption of rain-sheltering systems in kiwifruit orchards not only enhances fruit quality, but also significantly reduces disease incidence, making it a valuable practice for sustainable kiwifruit cultivation.

Moreover, shade management techniques are critical for protecting kiwifruit plants from sunburn damage, especially during hot summers or in regions with high solar radiation [112]. Shade nets or strategically planted trees provide partial shade, reducing the intensity of sunlight reaching the kiwifruit canopy and creating a more favorable microclimate within the orchard [113]. In a recent study, the impact of photo-selective nets (pearl, yellow, and grey) on *A. deliciosa* yield, fruit quality, and progression of bacterial kiwifruit canker was investigated. Compared to the control, the crop yield decreased by 40.3% under the three nets in 2020, and by 23.9% under the yellow and grey nets in 2021. In 2021, the yield and fruit grade of the plants under the pearl net were similar to those of the uncovered crops, with a higher overall fruit grade than those under the yellow and grey nets. Additionally, Psa progression decreased under the pearl nets compared to the control conditions, suggesting potential benefits for disease management [114]. Shade management through the use of photo-selective nets presents significant benefits for the cultivation of kiwifruit, particularly in regions with high solar radiation, such as the Mediterranean basin. A study conducted in Southern Italy on Jintao kiwifruit orchards exemplifies the positive impact of photo-selective nets on yield and fruit quality. The study showed that red nets exert a beneficial influence on critical kiwi production parameters, notably enhancing the net yield (56.84 kg·tree^−1^), dry matter content (19.61%), and total soluble solids (11.92 °brix), in comparison to open-field conditions or other colored nets [112]. These agronomic practices, when implemented effectively, can contribute to the sustainability and resilience of kiwifruit cultivation by minimizing environmental stressors and promoting optimal plant health and productivity.

### 5.3. Soil Conservation and Land-Use Planning Specific to Kiwifruit Farming

The impact of climate change on kiwifruit farming necessitates a holistic approach that integrates soil conservation and land-use planning strategies. Kiwifruit farming faces challenges, such as soil degradation, erosion, and changing precipitation patterns, which threaten its sustainability [38]. This note emphasized the importance of proactive measures to mitigate these challenges. The widespread adoption of conservation tillage methods, such as no-till, holds promise for preserving or even augmenting soil carbon levels, thus enhancing soil health and contributing significantly to soil conservation efforts [115]. No-till farming minimizes soil disturbance, preserves soil organic matter, and promotes soil health by creating a surface mulch layer that enhances microbial activity and nutrient cycling. It mitigates erosion, maintains soil structure, and improves water retention, leading to comparable or higher crop yields with lower input costs, particularly beneficial during drought periods [131]. Recent studies have demonstrated the adoption of cover cropping as a soil conservation practice. Cover cropping is an essential practice for maintaining soil health and preventing erosion in kiwifruit orchards. Cover crops are planted between kiwifruit rows during the off-season or as understory vegetation. These cover crops help to protect the soil surface from erosion by reducing the impact of raindrops and wind, as well as improving soil structure through root penetration and organic matter addition. Furthermore, cover crops contribute to weed suppression, nutrient cycling, and enhancing beneficial soil microbial communities [116,117,118]. Additionally, strategic land-use planning is crucial for optimizing kiwifruit farming practices while minimizing its environmental impacts. Zoning, land capability assessments, and agro-ecological planning can facilitate sustainable land use, by preserving biodiversity and ecosystem services [119,120,121]. By integrating soil conservation and land-use planning tailored to kiwifruit farming systems, the stakeholders can enhance resilience to climate change, ensure long-term sustainability, and foster a more resilient agricultural sector. The Yujiahe Catchment, located in Shaanxi Province, China, has seen a significant shift in land use, from traditional cereal crops to kiwifruit orchards, since 1990 [122]. This transition has made the area a key player in global kiwifruit production [123]. Despite this success, challenges like soil erosion and nutrient loss have emerged, stemming from factors such as steep slopes and concentrated rainfall. Previous studies have underscored the profound impact of land-use changes, from croplands to intensive orchards, on soil erosion, nutrient dynamics, and nitrate accumulation in deep soil profiles. While orchard development reduces erosion, it exacerbates phosphorus loss from fertilizer overuse and contributes to nitrate contamination of the groundwater, highlighting the imperative for sustainable land management practices in kiwifruit cultivation regions [124,125]. Soil conservation and efficient land-use planning are essential pillars of sustainable agriculture, particularly in kiwifruit cultivation. Upholding the soil quality and mitigating erosion are critical for maximizing the growth, productivity, and enduring sustainability of kiwifruit orchards. By prioritizing these practices, not only do we ensure the resilience of kiwifruit farming, but also contribute to the broader efforts of climate change adaptation and mitigation.

## 6. Case Studies and Success Stories of Kiwifruit Farming

### 6.1. Examples of Climate Change Adaptation in Kiwifruit Cultivation

Climate change poses significant challenges to kiwifruit cultivation, affecting various aspects of orchard management and fruit production. To effectively adapt to these challenges, kiwifruit growers have employed a range of innovative strategies aimed at mitigating the risks, enhancing resilience, and ensuring sustainable farming practices. Table 3 summarizes the adaptive strategies that have been employed in kiwifruit cultivation to combat the impacts of climate change, detailing the approaches taken and their effectiveness.

#### 6.1.1. Phenology Management

Climate change alters the timing of key phenological events in kiwifruit cultivation, such as the flowering of the fruit set and ripening. Rising temperatures, changing precipitation patterns, and extreme events can disrupt critical stages, from bud burst to flowering and fruiting, impacting fruit quality and yield [147,148]. Growers try to utilize adaptive planting strategies, such as adjusting planting dates and selecting the heat- and drought-tolerant cultivars *A. chinensis*, *A. arguta*, and *A. eriantha*, and synchronizing growth cycles with shifting climatic conditions [100,104,132]. Furthermore, the application of microclimate management strategies, such as shade netting, with shading percentages varying from 4% to 19% depending on the shade net type, effectively regulate orchard temperatures, reducing them by 1.3–7.6%. This method also increases moisture levels by 3.2–12.9%, alleviating heat stress and water stress during pivotal growth phases [112,133].

Changing the planting dates represents a proactive and flexible adaptation strategy that enables farmers to adapt to changing climatic conditions and minimize production risks. By incorporating local knowledge, scientific insights, and participatory approaches, agricultural communities can effectively harness the potential of adjusted planting dates to enhance agricultural resilience and ensure food security in a changing climate [38,149]. In addition, previous literature has concentrated on developing climate change-tolerant kiwifruit cultivars, primarily examining drought stress in *A. deliciosa*. Limited research has been conducted on the economically significant *Actinidia* species, such as *A. arguta* and *A. eriantha*. Korean researchers discovered that *A. arguta* and *A. eriantha* demonstrate superior drought tolerance when compared to *A. chinensis* and *A. deliciosa* [100]. In 2019, *A. deliciosa* cv. ‘Hayward’ experienced the most unfavorable conditions for yield and weight due to high temperatures (>30 °C) during the flowering phase. Consequently, researchers focused their efforts on addressing the challenges posed by elevated temperatures to mitigate their adverse impacts on kiwifruit production [150]. Previous studies have identified several kiwifruit species, including *A. rufa* and *A. chinensis deliciosa*, as possessing notably higher heat resistance levels. These findings underscore the potential of identified kiwifruit species as valuable sources of breeding cultivars better suited for warmer climatic conditions [39,134,135]. In conclusion, these works highlight the importance of proactive measures and continued research to improve kiwifruit’s resilience to climate change.

Recent research has underscored the importance of innovative orchard management techniques for kiwifruit growers facing climate change. Studies have highlighted the benefits of photo-selective nets for enhancing productivity and fruit quality. Research conducted in Portugal discovered that pearl nets create optimal microclimates and decrease bacterial canker progression. Although the nets did not significantly reduce the overall yield (only an 8.8% decrease), they enhanced the yield of extra grade kiwifruits compared to other types of nets [114]. In Southern Italy, white nets improved the dry matter and soluble solids by 16% and 9.3 °Brix, respectively, in ‘Hayward’ kiwifruit, while blue and grey nets had negative effects [136]. Additionally, over three years, red nets enhanced the fruit size and dry matter in mature vines, proving cost-effective [137]. Thus, adopting adaptive strategies and microclimate management techniques, such as photo-selective nets, are crucial for sustainable kiwifruit production amidst climate change.

#### 6.1.2. Water Resource Management

Changes in precipitation patterns and an increased frequency of extreme weather events, such as droughts or heavy rainfall, pose challenges for water management in kiwifruit orchards. Advanced irrigation technologies, including drip irrigation systems and soil moisture sensors, have been widely adopted to optimize water use efficiency and ensure adequate hydration for kiwifruit vines [138]. In response to the waterlogging caused by flooding or heavy rainfall, kiwifruit growers have adopted waterlogging-resistant cultivars. Furthermore, rainwater harvesting and storage systems have been implemented to supplement irrigation needs and mitigate water scarcity during dry periods [141].

A previous study suggested that timely and accurate access to drought information is crucial for successful kiwifruit cultivation, especially in regions prone to seasonal droughts, like southern China. The study indicated that the physiological indicators are more sensitive to soil moisture reduction compared to the morphological indicators, highlighting the importance of monitoring the physiological parameters for the early detection of drought stress in kiwifruit [151]. Recent studies have explored deficit drip irrigation as a water management strategy, applying irrigation below the full water requirement of kiwifruit crops, with numerous investigations conducted using similar approaches [105,109,139]. It involves intentionally providing less water to the plants than they would ideally require to fully satisfy their water needs. This approach aims to optimize water use efficiency and promote sustainable irrigation practices by strategically managing water resources [138]. Deficit drip irrigation has proven successful in improving kiwifruit quality and water productivity in South China’s humid areas, showcasing its effectiveness as a sustainable water management strategy [105,109,140]. These studies have implied that timely access to drought information and the adoption of deficit drip irrigation are crucial for successful kiwifruit cultivation, particularly in regions prone to seasonal droughts, like Southern China. These strategies optimize water use efficiency, mitigate drought effects, and inform effective irrigation management practices for kiwifruit growers and policymakers.

Waterlogging stress poses a significant challenge to kiwifruit cultivation, impacting plant growth, yield, and overall productivity. Grafting onto waterlogging-tolerant rootstocks presents a promising strategy with which to mitigate the adverse effects of waterlogging on kiwifruit plants. Recent research found that KR5 rootstock, derived from *A. valvata*, is more waterlogging-tolerant than the traditional *A. deliciosa* cv. ‘Hayward’ rootstock. Grafting the waterlogging-sensitive *A. deliciosa* cv. ‘Zhongmi 2’ scion onto KR5 improves the kiwifruit plant’s resilience under waterlogging stress, enhancing its photosynthetic efficiency and root activity, while reducing damage from reactive oxygen species. Using waterlogging-tolerant rootstocks like KR5 is crucial for kiwifruit production resilience [142]. Recent studies have also examined the different genes associated with waterlogging tolerance in kiwifruit [152,153,154,155,156]. Utilizing waterlogging-tolerant rootstocks, such as KR5, and understanding the genetic basis of waterlogging tolerance are essential steps towards enhancing kiwifruit production resilience in waterlogged conditions.

#### 6.1.3. Pest and Disease Control

Climate change has the potential to shift the distribution and prevalence of the pests and diseases impacting kiwifruit crops, potentially increasing pest pressure and disease outbreaks. This phenomenon poses significant challenges to global agriculture, as insect pests broaden their geographic expansion and alter their interactions [141]. Adaptive management strategies and enhanced research efforts are crucial to mitigate potential economic losses and ensure food security amidst changing climatic conditions. Integrated pest management (IPM) for kiwifruit in New Zealand, introduced from 1993 to 1997, replaced conventional pesticide use with monitoring, “soft” chemicals, and canopy management modifications. This approach led to cost savings, reduced health risks, and minimized environmental contamination, ensuring market access and consumer satisfaction while meeting evolving industry standards [143]. Additionally, the development of disease-resistant kiwifruit cultivars through breeding programs enhanced their resilience to climate-related pathogens. *P. syringae* pv. *actinidiae* biovar 3 (Psa3) causes kiwifruit bacterial canker, and has led to extensive damage to orchards worldwide since 2010. Cultivars like *A. chinensis* cv. ‘Hort16A’, initially less susceptible, have also been affected, underscoring the urgency of developing resistant varieties with which to combat this global threat [52,144]. Previous literature has highlighted the importance of cultivar resistance for combating *P. syringae* pv. *actinidiae* biovar 3 in kiwifruit orchards, with locally developed cultivars showing strong resistance, particularly those derived from *A. rufa* [145,146]. Utilizing resistant cultivars alongside the appropriate agrochemical controls may offer a viable strategy for achieving economically sustainable kiwifruit cultivation.

## 7. Methods

For a comprehensive review, a robust search of databases focusing on the impacts of climate change and the response strategies of kiwifruit producers was essential. Literature searches of several academic databases were conducted, including PubMed, Scopus, Web of Science, and Google Scholar. The keywords used in the search included “kiwifruit”, “climate change”, “mitigation strategy”, and “adaptation strategy.” Boolean operators were employed to refine the search criteria. This review process encompassed the literature from 1998 to 2024, with a preference for recent publications to ensure the information was current.

## 8. Conclusions and Future Perspectives

This review paper offers comprehensive insights into the diverse impacts of climate change on kiwifruit production, and presents a spectrum of adaptation and mitigation strategies with which to address these challenges. With global temperatures on the rise and precipitation patterns undergoing shifts, kiwifruit-producing regions are increasingly vulnerable to extreme weather events and changing environmental conditions, posing significant economic and social hurdles for farmers and agricultural communities reliant on kiwifruit cultivation. To counteract these challenges, a suite of adaptation and mitigation measures are proposed, ranging from the development of resilient kiwifruit cultivars to the implementation of sustainable agronomic practices and soil conservation efforts.

However, certain mitigation strategies, such as land-use changes and excessive irrigation to combat drought, have inadvertently led to nitrogen accumulation and environmental degradation. Additionally, challenges persist to developing disease-resistant kiwifruit cultivars, highlighting the ongoing need for research and the adoption of locally developed varieties exhibiting strong resistance. Therefore, investment in research and development initiatives should further support the breeding of climate-resilient kiwifruit varieties and the refinement of agronomic practices. This will ultimately enhance the resilience and sustainability of kiwifruit farming systems amidst a changing climate.

Our study contributes to the existing body of knowledge by presenting detailed approaches that demonstrate successful adaptation and mitigation strategies. These insights offer practical recommendations for future research aimed at developing kiwifruit cultivation practices in the context of climate change.

## Figures and Tables

**Table 1:** Impact of climate change on kiwifruit production.

S. No.	Global Climate Change or Its Effect	Specific Impact on Kiwifruit Production	Location	Study Details or Observations	References
1	Global temperature rise	Decreased winter chilling, leading to reduced viability for ‘Hayward’ kiwifruit.	Te Puke, New Zealand	Production becoming non-viable by century’s end due to insufficient chilling.	[37]
2	Temperature fluctuations	Impact on photosynthesis and vine health; severe leaf damage at high temperatures.	Global	Photosynthetic rate impairment and irreversible leaf damage at 52 °C.	[39]
3	Frosting events	Increased risk of spring frost damage.	Boseong, Korea	Rising risks due to earlier frost dates and accelerated bud burst.	[41,42]
4	Droughts and floods	Disruption in photosynthesis and fruit quality; increased abscisic acid levels during droughts.	New Zealand, Global	Water stress management crucial; impact of droughts and floods on yield and fruit quality.	[43,44]
5	Typhoons and strong winds	Structural damage to plants, disrupted fruit setting, and lowered pollination rates.	Global	Strong wind causes significant orchard damage and affects various growth stages.	[42,45]
6	Phenological changes	Increase in flowering days; shifts in flowering and fruiting periods.	Korea, New Zealand	Northward expansion and earlier flowering of ‘Haegeum’; less winter chilling affecting ‘Hayward’ in Te Puke.	[37,46]
7	Shifts in suitable growing regions	Expansion of kiwifruit cultivation to new areas due to warming temperatures.	Korea	Cultivation spreads to new regions, like Sacheon and Jeju Island.	[47]
8	Physiological impacts	Altered photosynthesis and respiration rates due to temperature and humidity stress.	Global	Need for water management to sustain photosynthetic activity; respiration inhibited above 44.5 °C.	[39,48,49]
9	Increased pests and diseases	Heightened prevalence of Psa and susceptibility to new pathogens.	Global	Rising temperatures foster conditions favorable for pathogens like Psa.	[50,51,52,53,54,55,56]

**Table 2:** Adaptation and mitigation strategies for kiwifruit cultivation: climate-resilient cultivars, agronomic practices, soil conservation, and land-use planning.

S. No.	Category	Strategy/Approach	Description	References
1	Breeding and Selection	Climate-resilient cultivars	Developing kiwifruit cultivars with heat tolerance, drought resistance, and disease resilience to withstand extreme weather events.	[39,100,101,102,103]
		High-temperature tolerance genes	Identifying heat shock transcription factor (Hsf) genes in *A. chinensis* and *A. eriantha* to breed heat-stress-resistant cultivars.	[104]
2	Agronomic Practices	Irrigation management	Utilizing drip and sprinkler irrigation to minimize water loss and optimize water use efficiency based on soil moisture levels, weather conditions, and plant growth.	[105,106]
		Mulching	Applying organic or synthetic mulch to conserve soil moisture, suppress weeds, moderate soil temperatures, and prevent erosion.	[107,108]
		Rain-shelter cultivation system	Implementing rain-shelter systems to protect kiwifruit flowers from heavy rainfall, reduce disease incidence, and improve fruit quality.	[109,110,111]
		Shade management	Using shade nets or strategically planted trees to reduce sunburn damage and create favorable microclimates within orchards.	[112,113,114]
3	Soil Conservation and Land-Use Planning	Conservation tillage (no-till farming)	Minimizing soil disturbance to preserve organic matter, enhance microbial activity, improve water retention, and reduce erosion.	[115]
		Cover cropping	Planting cover crops to protect soil from erosion, improve soil structure, suppress weeds, and enhance nutrient cycling and beneficial soil microbial communities.	[116,117,118]
		Strategic land-use planning	Implementing zoning, land capability assessments, and agro-ecological planning to optimize land use, preserve biodiversity, and minimize environmental impacts.	[119,120,121]
		Managing shifts in land use	Transitioning from traditional crops to kiwifruit orchards, addressing challenges like soil erosion and nutrient loss, and promoting sustainable land management.	[122,123,124,125]

**Table 3:** Adaptive strategies for kiwifruit cultivation in response to climate change: phenology management, water resource management, and pest and disease control.

S. No.	Category	Strategy/Approach	Description	References
1	Phenology management	Adaptive planting	Adjust planting dates and select cultivars tolerant to heat and drought to match growth cycles with new climatic conditions.	[132]
		Microclimate management	Use of shade netting to manage orchard temperatures and moisture, reducing heat and water stress.	[133]
2	Breeding	Selection of climate-resilient cultivars	Develop and use kiwifruit cultivars with improved drought and heat tolerance, like *A. arguta* and *A. eriantha*.	[100]
		Heat-resistant cultivars	Focus on species with higher heat resistance for breeding, such as *A. rufa* and *Jintao* cultivars.	[39,134,135]
3	Orchard management	Photo-selective nets	Implement pearl photo-selective nets in orchards to create optimal microclimates, enhance productivity, and manage diseases like bacterial kiwifruit canker.	[114]
		Hail netting	Use colored hail netting to optimize fruit quality and orchard productivity.	[136,137]
4	Water management	Advanced irrigation technologies	Use drip irrigation systems and soil moisture sensors to optimize water use efficiency.	[138]
		Deficit drip irrigation	Apply less water than full crop requirements to improve water productivity and kiwifruit quality.	[105,109,139,140]
		Rainwater harvesting	Implement systems to collect and store rainwater, supplementing irrigation needs during dry periods.	[141]
5	Waterlogging mitigation	Grafting onto waterlogging-tolerant rootstocks	Use KR5 rootstock for better resilience of kiwifruit plants under waterlogging conditions.	[142]
6	Pest and disease control	Integrated pest management (IPM)	Implement IPM strategies to replace conventional pesticide use, reducing health risks and environmental impact.	[143]
		Development of disease-resistant cultivars	Breed and utilize kiwifruit cultivars resistant to diseases like Psa3, enhancing resilience to climate-related pathogens.	[52,144,145,146]
